# Paternal Age and Offspring Congenital Heart Defects: A National Cohort Study

**DOI:** 10.1371/journal.pone.0121030

**Published:** 2015-03-25

**Authors:** Xiu Juan Su, Wei Yuan, Guo Ying Huang, Jørn Olsen, Jiong Li

**Affiliations:** 1 Clinical and Translational Research Center, Shanghai First Maternity and Infant Hospital, Tongji University School of Medicine, Shanghai, China; 2 Section for Epidemiology, Department of Public health, Aarhus University, Aarhus, Denmark; 3 NPFPC Laboratory of Contraception and Devices, Shanghai Institute of Planned Parenthood Research, Shanghai, China; 4 Institute of Reproduction and Development, Fudan University, Shanghai, China; 5 Children‘s Hospital of Fudan University, Shanghai, China; CHU Sainte Justine and University of Montreal, CANADA

## Abstract

Paternal age has been associated with offspring congenital heart defects (CHDs), which might be caused by increased mutations in the germ cell line because of cumulated cell replications. Empirical evidences, however, remain inconclusive. Furthermore, it is unknown whether all subtypes of CHDs are affected by paternal age. We aimed to explore the relationship between paternal age and the risk of offspring CHDs and its five common subtypes using national register data in Denmark. A total of 1 893 899 singletons born in Denmark from 1977 to 2008 were included in this national-based cohort study. Cox’s proportion hazards model with robust sandwich estimate option was used to estimate the hazards ratio (95% confidence interval) for the associations between paternal age and all CHDs, as well as subtypes of CHDs (patent ductus arteriosus (PDA), ventricular septal defect (VSD), atrial septal defect (ASD), tetralogy of fallot (TOF) and coarctation of the aorta (CoA)). We did not observe an overall association between paternal age and offspring CHDs. However, compared to the paternal age of 25–29 years, paternal age of older than 45 years was associated with a 69% increased risk of PDA (HR45+ = 1.69, 95%CI:1.17–2.43). We observed similar results when subanalyses were restricted to children born to mothers of 27–30 years old. After taking into consideration of maternal age, our data suggested that advanced paternal age was associated with an increased prevalence of one subtype of offspring congenital heart defects (CHDs), namely patent ductus arteriosus (PDA).

## Introduction

Congenital heart defects (CHDs) are the most common congenital malformations, affecting 5–10 per 1000 live births [1–3] but the etiology of CHDs remains to be identified. Advanced parental age, race, ethnicity, and chemical agents have been proposed as potential risk factors [4–6]. It was also reported that there is gender difference in the prevalence of CHDs [7]. Postponement of maternal age for first child birth could be observed in many countries since 1970s [8]. Similar to maternal age, there has been a 20% increase for children born to fathers over 35 years old in the past 20 years in USA, and the paternal childbearing peak years has changed from 25–29 years to 30–34 years since 2008 [9]. The average paternal age at child birth has also exceeded 40 years old and more than 40% of fathers were 40–49 years old at child birth in 2006 in Canada [10].

It has been speculated that paternal age was critically significant for gene mutation since 1955 [11], and it was reported the diversity in mutation rates of single nucleotide polymorphisms is dominated by paternal age at conception of the child by sequencing the entire genomes of 78 Icelandic parent–offspring trios at high coverage [12]. Some behavior factors may interact with genetic factors related to the paternal age on the development of offspring, such as smoking and alcohol etc. [13,14]. Paternal age has been related to an increased prevalence of different types of birth defects, such as abnormal limbs and neural tube defects [15,16]. Recently, advanced paternal age is proposed to be a risk factor of the heart malformation in the offspring in some studies [17–20], but not in the others [21–23]. Two studies have examined the subtypes of CHDs with inconsistent findings. Olshan et al observed increased risks of ventricular septal defect (VSD) and atrial septal defect (ASD) in children born to fathers younger than 20 years old or older than 35 years old, while an increased risk of patent ductus arteriosus (PDA) was found in children born to fathers older than 35 years old [18]. Bassili et al reported that paternal age of older than 40 years was a risk factor for VSD, but not for ASD [24]. Due to the close correlation between paternal age and maternal age, it may be misleading to examine the effects of paternal age without taking maternal age into consideration.

Considering high prevalence of CHDs and limited epidemiological evidences related to paternal age effect on specific types of CHDs, we conducted this national-based cohort study to explore the association between paternal age and overall risk of CHDs in the offspring, including the five major subtypes of CHDs.

## Materials and Methods

The population-based cohort study was based on secondary data and all analyses were done at the secure platform of Denmark Statistics, using encrypted identification numbers without any access to personal identification numbers. The study was approved by the Danish Data Protection Agency (J NR. 2008–41–2680) and the local ethics committee in Central Denmark Region (VEK, case number M-201000252).

Every live birth was assigned a unique personal civil registration number, which can be used to link the information at individual level among all national registries. Data was retrieved from several Danish registries, including the Danish Medical Birth Registry [25], the Danish Register of Congenital Heart Disease [26], the Danish National Patient Register [27], and the Danish Civil Registration System [28].

### Study population

We identified 1 927 278 liveborn singletons in Denmark between January 1, 1977 and December 31, 2008 from the Danish Medical Birth Registry. We excluded 33 379 (1.73%) children with no information on the mother and the father. A total of 1 893 899 singletons remained in the study.

### Exposure and outcome

We used the Danish Civil Registration System to identify the paternal and maternal birth date, and then calculated their ages at the time of delivery [28]. The paternal age was treated as continuous variable and also categorized into seven groups (<20, 20–24, 25–29, 30–34, 35–39, 40–44, 45+ years).

CHDs were obtained from the Danish Register of Congenital Heart Disease, which contains data on CHDs from 1963 to the present [26]. Diseases were coded by the 8^th^ Revision of the International Classification of Diseases (ICD-8) until the end of 1993 and thereafter the 10^th^ Revision(ICD-10) [27]. ICD-8 codes for CHDs were 746–747, except for 746.7, 746.9 and 747.5–747.9; and ICD-10 codes for CHDs were Q20-Q26, except for Q20.9, Q21.9, Q24.9, Q25.9, Q26.5, Q26.6 and Q26.9 [29]. Children with more than one type of CHDs were categorized according to first CHDs diagnosis in the registry.

The most five common CHDs included patent ductus arteriosus (PDA, codes 747.0(ICD-8) and Q25.0 (ICD-10)), ventricular septal defect (VSD, codes 746.3 (ICD-8) and Q21.0 (ICD-10)), atrial septal defect (ASD, codes 746.4 (ICD-8) and Q21.1 (ICD-10)), tetralogy of fallot (TOF, codes 746.2 (ICD-8) and Q21.3 (ICD-10)) and coarctation of the aorta (CoA, codes 747.1 (ICD-8) and Q25.1 (ICD-10)).

### Statistical analysis and potential confounders

The statistical software package SAS version 9.2 (SAS Institute Inc., Cary, NC, USA) was used for data analysis. CHDs are malformations present at birth but often identified later on life, therefore the length of follow-up has to be taken into consideration. Cox’s proportion hazards model was used to estimate hazards ratios (HRs) and corresponding 95% confidence interval (95%CI). Follow up start from the birth date of the children, ended on the date of CHDs diagnosis, death, emigration, or the end of follow up on December 31, 2010, whichever came first. We observed that the hazards of offspring CHDs for different paternal age groups are proportional over time (S1 Fig.). The robust sandwich estimate option was used to deal with the potential cluster effect caused by siblings.

The age group of 25–29 years was treated as the reference group because it might be expected to be the optimal childbearing age period [30,31]. Since maternal age was non-linear associated with CHDs [32], it was included in the model both linear and quadratic terms in 1 year unit to secure a better fit of the model [33]. Considering different parental age pattern might bias the correlation between paternal age and prevalence of CHDs [34], the absolute value of parental age difference was also included in the model (<5, 5–9, ≥10 years). Genetic factor was proposed be a risk factor of CHDs. Family history of CHDs as a consequence was included in the model, identified by the fact that one of close relatives was diagnosed as CHDs, including father, mother, sibling, grandmother and grandfather by the Danish Register of Congenital Heart Disease. Because of its effect on the fetus development [35], maternal infection during pregnancy was also included in the model identified by the Danish National Patient Register. For the overall prevalence of CHDs, the analysis was carried out in two models. In the first model, linear and quadratic terms of maternal age, parental age difference (<5, 5–9, ≥10 years), gender of the children (girl, boy), parity (1^st^, 2^nd^, 3^rd^ and higher), calendar year of the children (1977–1980, 1981–1985, 1986–1990, 1991–1995, 1996–2000, 2001–2005, 2006–2008) were included (Model 1). In the second model, in addition to those covariates in model 1, family history of CHDs and maternal infection during pregnancy were further added (Model 2). Only model 2 was used to estimate adjusted HRs regarding to subtypes of CHDs.

Maternal age was closely correlated with paternal age (r = 0.68 in this data set). Thus we performed subanalyses to estimate the HRs of CHDs and its subtypes by restricting to children with 27–30 years of maternal age (n = 590 455), which could reduce the confounding by maternal age effect. In order to avoid potential sparse data bias, the paternal age was re-categorized into 5 groups by combining the <20 years group and 20–24 years group into one group (≤24 years), so was the 40–44 years group and 45+ years group (40+ years). We performed sensitivity analyses among children without (n = 1 849 348) or with CHDs family history (n = 44 551) to partly disentangle genetic effects from paternal age effects. Because data on the maternal social status and maternal smoking was available after 1981 and 1991 respectively, we conducted subanalyses among children born after 1981 adjusted for the covariates in model 2, and additionally for maternal social status at birth (not in the labor, blue collar, white collar, higher position, missing) (Model 3). Then we performed subanalyses among children born after 1991 adjusted for the covariates in model 3, and additionally for maternal smoking status (yes, no) (Model 4). We also did stratified analyses by child’s gender to explore the gender difference as reported in previous studies [7].

## Results

Table 1 shows the sociodemographic characteristics among different paternal age groups. About 63.69% children were born to fathers at 25–34 years of age at birth. Children born to older fathers were likely to have older mothers. The proportion of maternal infection during pregnancy was higher both in younger and advanced paternal age group. Children born to younger fathers were more likely to have smoking mothers who were also not in the labor market.

**Table 1:** Characteristics between different paternal age groups at birth (n/%).

	Paternal age Mean ± SD	<20	20–24	25–29	30–34	35–39	40–44	45+
Maternal age								
≤26	27.4 ± 4.4	7 255(97.9)	147 313(91.5)	312 263(56.5)	118 727(18.2)	28 588(8.2)	7 422(6.1)	3 083(6.3)
27–30	31.3 ± 4.1	111(1.5)	10 678(6.6)	196 263(35.5)	285 051(43.6)	74 417(21.3)	17 305(14.3)	6 630(13.6)
≥31	35.8 ± 5.1	45(0.6)	3 053(1.9)	43 825(7.9)	250 156(38.3)	246 657(70.5)	96 094(79.5)	38 963(80.1)
Gender								
Boy	31.6 ± 5.8	3 739(50.5)	83 099(51.6)	283 347(51.3)	335 799(51.4)	179 219(51.3)	62 195(51.5)	24 824(51.0)
Girl	31.6 ± 5.8	3 672(49.6)	77 945(48.4)	269 004(48.7)	318 135(48.7)	170 443(48.8)	58 626(48.5)	23 852(49.0)
Parity								
1	29.9 ± 5.7	6 887(92.9)	124 790(77.5)	324 968(58.8)	257 801(39.4)	102 421(29.3)	35 103(29.1)	16 847(34.6)
2	32.3 ± 5.2	438(5.9)	31 476(19.5)	189 333(34.3)	292 184(44.7)	146 262(41.8)	45 069(37.3)	16 832(34.6)
3+	35.1 ± 5.5	80(1.1)	4 720(2.9)	37 915(6.9)	103 821(15.9)	100 885(28.9)	40 617(33.6)	14 965(30.7)
Missing	32.2 ± 7.7	6(0.1)	58(0.0)	135(0.0)	128(0.0)	94(0.0)	32(0.0)	32(0.1)
Family history of CHDs								
Yes	31.6 ± 5.8	279(3.8)	4 662(2.9)	12 837(2.3)	14 527(2.2)	8 233(2.4)	2 826(2.3)	1 187(2.4)
No	31.4 ± 6.0	7 132(96.2)	156 382(97.1)	539 514(97.7)	639 407(97.8)	341 429(97.6)	117 995(97.7)	47 489(97.6)
Maternal infection								
Yes	31.6 ± 5.8	353(4.8)	5 992(3.7)	16 937(3.1)	18 910(2.9)	10 498(3.0)	3 846(3.2)	1 615(3.3)
No	31.5 ± 6.0	7 058(95.2)	155 052(96.3)	535 414(96.9)	635 024(97.1)	339 164(97.0)	116 975(96.3)	47 061(96.7)
Maternal social status								
Not in the labor	31.4 ± 6.8	3 856(67.5)	40 693(32.5)	84 562(18.2)	86 658(14.9)	52 747(16.3)	22 837(20.2)	11 909(26.4)
Blue collar	33.8 ± 6.0	66(1.2)	2 373(1.9)	13 264(2.9)	22 685(3.9)	16 526(5.1)	6 891(6.1)	3 144(7.0)
White collar	31.1 ± 5.6	1 302(22.8)	52 211(41.7)	173 907(37.3)	180 527(31.0)	91 089(28.2)	29 952(26.5)	10 894(24.2)
Higher position	32.5 ± 5.3	305(5.3)	28 568(22.8)	190 445(40.9)	288 128(49.5)	160 440(49.6)	52 281(46.2)	18 266(40.5)
Missing ^a^	32.4 ± 7.1	183(3.2)	1 472(1.2)	3 680(0.8)	4 507(0.8)	2 806(0.9)	1 224(1.1)	855(1.9)
Maternal smoking								
Yes	32.7 ± 5.6	1 257(43.7)	21 972(35.5)	65 951(23.2)	74 345(18.1)	42 811(17.8)	16 131(18.9)	6 764(19.6)
No	31.7 ± 6.0	1 326(46.1)	34 123(55.1)	193 037(69.0)	297 235(72.2)	172 142(71.6)	59 402(69.7)	23 835(69.1)
Missing ^b^	30.7 ± 5.8	294(10.2)	5 802(9.4)	24 812(8.7)	39 882(9.7)	25 547(10.6)	9 735(11.4)	3 908(11.3)

^a^No information for children born before 1981

^b^no information for children born before 1991.

A total of 15 216 (0.80%) CHDs were ascertained from 1 893 899 live births. Compared to the reference group, there was no difference in the prevalence of overall CHDs in different paternal age groups by model 1 and model 2 (Table 2). The results for each subtype of CHDs are shown in S1 Table. We observed paternal age of older than 45 years of age had an increasing risk of PDA in the offspring (HR_45+_ = 1.69, 95%CI: 1.17–2.43) (Table 3). No statistically significant associations were observed between paternal age and prevalence of ASD, VSD, TOF, and CoA.

**Table 2:** The incidence rates and hazards ratios of congenital heart defects according to paternal age groups.

Paternal age	No. of CHDs/No. of birth	Incidence rate1/1000 person years)	Crude HR	HR^model 1^(95%CI)	HR^model 2^ (95%CI)
<20	61/7 411	47.91	0.89	0.96(0.74–1.25)	0.99(0.76–1.28)
20–24	1 257/161 044	48.06	0.85	0.98(0.92–1.05)	1.00(0.93–1.07)
25–29	4 364/552 351	54.06	1.0(Ref)	1.00	1.00
30–34	5 123/653 934	62.16	1.18	0.97(0.93–1.02)	0.96(0.91–1.00)
35–39	2 858/349 662	67.78	1.37	0.97(0.91–1.03)	0.93(0.87–0.99)
40–44	1 076/120 821	70.48	1.57	1.01(0.93–1.10)	0.96(0.87–1.05)
45+	477/48 676	68.85	1.68	1.07(0.95–1.23)	1.00(0.88–1.14)

^Model 1^Adjustment for linear and quadratic terms of maternal age, parental age difference, gender of the child, parity, and calendar year of child birth; ^Model2^Adjustment for Model 1+ family history of CHDs and maternal infection during pregnancy.

**Table 3:** Hazards ratios of the five common subtypes of congenital heart defects by paternal age groups.

	PDA(n = 1 748)		ASD(n = 2 543)		VSD(n = 3 628)		TOF(n = 365)		CoA(n = 458)	
	HR^a^	HR^b^	HR^a^	HR^b^	HR^a^	HR^b^	HR^a^	HR^b^	HR^a^	HR^b^
<20	1.24	1.11(0.56–2.21)	0.68	0.71(0.33–1.52)	0.93	0.99(0.59–1.65)	0.68	0.80(0.11–6.08)	-^c^	-^c^
20–24	0.92	0.98(0.80–1.20)	0.80	0.94(0.79–1.12)	0.84	0.98(0.85–1.12)	1.16	1.41(0.92–2.16)	0.64	0.83(0.53–1.29)
25–29	1.00	1.00	1.00	1.00	1.00	1.00	1.00	1.00	1.00	1.00
30–34	1.26	1.09(0.95–1.25)	1.31	1.03(0.92–1.15)	1.14	0.93(0.85–1.02)	1.20	0.94(0.70–1.27)	1.31	1.00(0.79–1.27)
35–39	1.47	1.12(0.93–1.34)	1.58	1.02(0.89–1.18)	1.30	0.89(0.79–1.01)	1.11	0.70(0.48–1.03)	1.21	0.75(0.54–1.04)
40–44	1.77	1.26(0.97–1.63)	1.86	1.10(0.90–1.35)	1.37	0.85(0.71–1.02)	1.98	1.05(0.63–1.77)	1.17	0.65(0.38–1.10)
45+	2.28	1.69(1.17–2.43)	1.75	1.06(0.78–1.43)	1.30	0.80(0.61–1.06)	1.84	0.87(0.42–1.79)	1.98	1.03(0.51–2.09)

^a^Crude hazards ratios

^b^Adjustment for linear and quadratic terms of maternal age, parental age difference, gender of the child, parity, family history of CHDs, calendar year of childbirth, and maternal infection during pregnancy

^c^No cases.

The sub-analyses results restricted to children born to mothers of 27–30 years were shown in Table 4. Children born to fathers older than 40 years had an increasing prevalence of PDA (HR_40+_ = 1.60, 95%CI: 1.10–2.32). We found similar results when we restricted analyses to children without CHDs family history (S2 Table). When analyses were restricted to children with CHDs family history, we observed increased risks for both overall and subtypes of CHDs (S3 Table). Similar to the main analyses, we only observed an increasing risk of PDA for paternal age of older than 45 years of age when analyses were restricted to children born after 1981 by model 3 (data not shown). However, the HR of PDA tended to be insignificant when restricting children born after 1991 by model 4 (HR_45+_ = 1.33, 95%CI: 0.95–1.84) (data not shown). We did not find the gender difference of the associations between paternal age and CHDs (data not shown).

**Table 4:** Hazards ratios of the congenital heart defects by paternal age with maternal age of 27–30 years(n = 590 455).

	PDA(n = 507)		ASD(n = 767)		VSD(n = 1 104)		TOF(104)		CoA(n = 152)		CHDs(n = 4 606)	
	HR^a^	HR^b^	HR^a^	HR^b^	HR^a^	HR^b^	HR^a^	HR^b^	HR^a^	HR^b^	HR^a^	HR^b^
<24	1.21	1.34(0.75–2.42)	0.53	0.60(0.30–1.21)	1.08	1.19(0.79–1.78)	0.94	0.99(0.24–4.08)	1.77	1.99(0.78–4.97)	0.95	1.04(0.84–1.28)
25–29	1.00	1.00	1.00	1.00	1.00	1.00	1.00	1.00	1.00	1.00	1.00	1.00
30–34	1.01	0.99(0.81–1.22)	1.03	1.01(0.86–1.18)	0.95	0.93(0.81–1.06)	1.02	1.04(0.68–1.60)	1.13	1.07(0.75–1.53)	0.96	0.94(0.88–1.00)
35–39	1.07	0.95(0.71–1.28)	1.23	1.09(0.87–1.36)	1.07	0.95(0.78–1.15)	0.63	0.60(0.28–1.28)	1.20	1.05(0.62–1.79)	1.08	0.96(0.87–1.06)
40+	1.78	1.60(1.19–2.32)	1.05	0.95(0.65–1.39)	0.94	0.85(0.61–1.17)	1.46	1.38(0.58–3.26)	1.18	1.06(0.45–2.48)	1.19	1.07(0.93–1.24)

^a^Crude HRs

^b^Adjustment for calendar year of the birth, gender of the child, parity, family history of CHDs, and maternal infection during pregnancy.

## Discussion

In this study, we did not find an overall association between paternal age and prevalence of offspring’s combined CHDs. No associations were observed between paternal age and prevalences of ASD, VSD, TOF and CoA in the offspring. However, we observed an increased prevalence of PDA associated with paternal age of older than 45 years.

The biological mechanism behind the association between paternal age and offspring CHDs is not clear but several hypotheses have been proposed. First, increasing male age is associated with a deterioration of semen quality, including volume, sperm concentration, motility and morphology [36]. Second, there are increasing mutations as increasing germ cell replications error following the advanced paternal age [37]. In addition, older males have increased sperm DNA damages associated with alkali-labile sites or single-strand DNA breaks. DNA damages in sperm can be converted to chromosomal aberrations and gene mutations after fertilization, increasing the risk of the developmental defects and genetic diseases among offspring [38]. Third, exposure to environmental risk factors may be linked to sperm genetic change because of accumulated effects. This was elaborated both in animal models and the epidemiologic studies [39,40]. Forth, the present explanation for the young paternal age effect is that more unhealthy lifestyle among young couples may be prevalent, such as smoking and alcohol use [16].

However, our findings on no overall association between paternal age and prevalence of CHDs, which are consistent with some previous studies [41,42], but not the others [17,20,24]. One reason could be that maternal age was not well taken into consideration in previous studies. We observed that children born to younger fathers were more likely to have mothers with smoking habits, infections in pregnancy and not in the labor market. Similar to our study, young paternal age was observed to be associated with CHDs found in a Norwegian study, and this could be attributable to unhealthy life styles, as suggested by the authors [16]. In addition, the Danish Register of Congenital Heart Disease, which is used to identify the cases in the present study, has excluded children diagnosed with isolated ASD, VSD or PDA before the age of 2 months if there were no subsequent records of CHDs in the DNPR [43], which could attenuate the overall association.

There has been very limited research on the association between paternal age and subtypes of CHDs [18,23,24]. Mutations in three transcription factor genes, TBX5, NKX2–5 and GATA4 have been reported, which can induce secundum ASDs and mutation in CRELD1, encoding a cell adhesion molecule causing AVSDs [44]. It has been suggested that mutation TFAP2B causes Char syndrome, which is a familial form of PDA [45]. All these studies indicate that different underlying biological mechanisms among different subtypes of CHDs. However, there has been no research on the association between these gene mutations and paternal age. We found advanced paternal age was associated with increasing prevalence of the PDA. This was consistent with a previous study in which the authors found increasing prevalence of PDA with increasing paternal ages [18]. We did not find an association between paternal age and prevalence of the other four subtypes of CHDs, which is partly supported by three previous studies [23,24,46]. In addition, when restricted analyses to children born to mother with 27–30 years of age, we found an increased prevalence of TOF and CoA among children with advanced paternal age, as well an increased prevalence of PDA, VSD and CoA among children with younger paternal age. However, all of these associations were statistically insignificant, which might be due to small sample size.

To the best of our knowledge, the present study has the largest sample size based on national population data, which allowed us to evaluate the independent effect of paternal age on subtypes of CHDs by taking the effect of maternal age into consideration. However, our findings should be interpreted with caution in the light of following limitations: (1) we do not have data on paternal lifestyle factors. (2) Because of lacking of standard transformation between the ICD-8 and ICD-10, we could not analyze more subtypes of CHDs during this period. (3) We have data on live births only, not data on the stillbirth, spontaneous abortion, and other pregnancy terminations, which, however, might lead to underestimations of the associations between paternal age and offspring CHDs.

To conclude, after taking into consideration of maternal age and other potential confounders, we observed that there was no association between paternal age and prevalence of offspring overall CHDs. However, our data suggested that advanced paternal age may be associated with an increased prevalence of a subtype of CHDs, namely PDA.

## Supporting Information

S1 Fig Test the assumption of Cox’s proportional hazards model. Hazards in different paternal age groups compared with reference age group (25–29 years) are almost proportional over time.
(TIF)

S1 Table Incidence Rate of subtypes of CHDs in different paternal age groups. (DOCX)

S2 Table Adjusted hazards ratio of CHDs and its five common subtypes among children without CHDs family history by different paternal age groups. (DOCX)

S3 Table Adjusted hazards ratio of CHDs and its five common subtypes among children with CHDs family history by different paternal age groups. (DOCX)
