# Supplementary figures and images for: Paternal Age and Offspring Congenital Heart Defects: A National Cohort Study
Source: PLoS One. 2015 Mar 25;10(3):e0121030. doi: 10.1371/journal.pone.0121030 (PMC4373953; doi:10.1371/journal.pone.0121030)

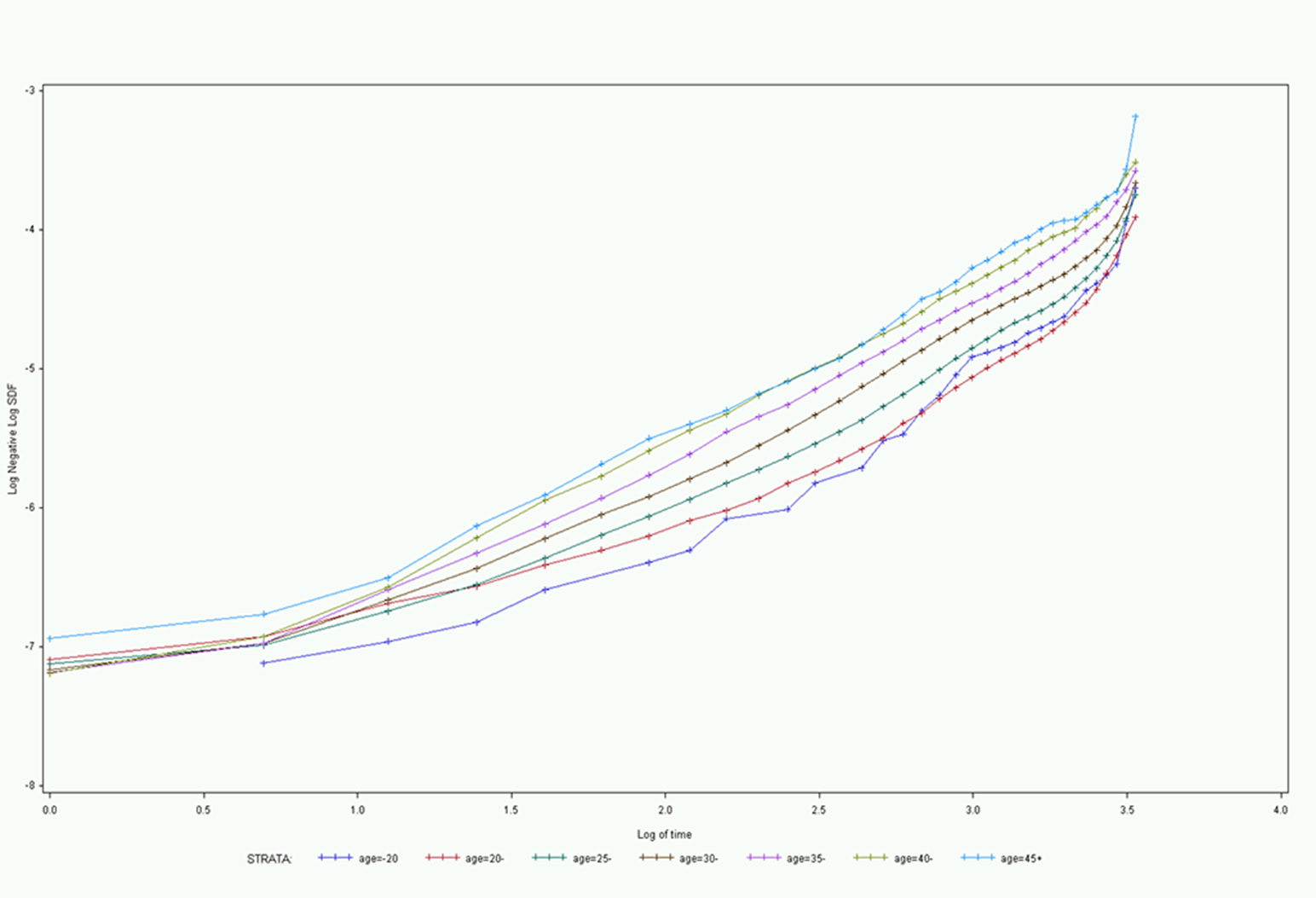

Supplement: S1 Fig — Hazards in different paternal age groups compared with reference age group (25–29 years) are almost proportional over time. (TIF) [file pone.0121030.s001.tif]
