# Supplementary material for: Paternal Age and Offspring Congenital Heart Defects: A National Cohort Study
Source: PLoS One. 2015 Mar 25;10(3):e0121030. doi: 10.1371/journal.pone.0121030 (PMC4373953; doi:10.1371/journal.pone.0121030)
Supplement: S1 Table — (DOCX) [file pone.0121030.s002.docx]

**S1 Table. Incidence Rate (IR^*^) of subtypes of CHDs** **i****n different paternal age groups (1/100,000 person years).**

| Paternal age | PDA(n=1 748) | | ASD(n=2 543) | | VSD(n=3 628) | | TOF(365) | | CoA(n=458) | |
| --- | --- | --- | --- | --- | --- | --- | --- | --- | --- | --- |
|  | No. ^+^ | IR | No. | IR | No. | IR | No. | IR | No. | IR |
| <20 | 9 | 5.82 | 7 | 4.53 | 16 | 10.34 | 1 | 0.65 | 0 | 0.00 |
| 20-24 | 143 | 4.27 | 178 | 5.31 | 313 | 9.34 | 37 | 1.10 | 30 | 0.90 |
| 25-29 | 466 | 4.56 | 672 | 6.58 | 1 091 | 10.68 | 99 | 0.97 | 136 | 1.33 |
| 30-34 | 593 | 5.64 | 888 | 8.44 | 1 227 | 11.66 | 124 | 1.18 | 175 | 1.66 |
| 35-39 | 333 | 6.46 | 517 | 10.02 | 661 | 12.81 | 57 | 1.10 | 76 | 1.47 |
| 40-44 | 133 | 7.76 | 202 | 11.78 | 229 | 13.36 | 34 | 1.98 | 24 | 1.40 |
| 45+ | 71 | 10.04 | 79 | 11.17 | 91 | 12.87 | 13 | 1.84 | 17 | 2.40 |

*IR: Incidence Rate; +: Number of cases.
