# Supplementary material for: Paternal Age and Offspring Congenital Heart Defects: A National Cohort Study
Source: PLoS One. 2015 Mar 25;10(3):e0121030. doi: 10.1371/journal.pone.0121030 (PMC4373953; doi:10.1371/journal.pone.0121030)
Supplement: S2 Table — (DOCX) [file pone.0121030.s003.docx]

**S2 Table.** **Adjusted^a^ hazards ratio of CHDs and its five common subtypes among children without CHDs family history by different paternal age groups**

| Paternal age | CHDs(n=14 228) | PDA(n=1 623) | ASD (n=2 370) | VSD (n=3 447) | TOF(n=336) | COA(n=429) |
| --- | --- | --- | --- | --- | --- | --- |
| <20 | 0.86(0.65-1.15) | 0.67(0.27-1.65) | 0.53(0.1-1.31) | 1.05(0.63-1.75) | 0.86(0.11-6.59) | b |
| 20-24 | 0.96(0.90-1.03) | 0.99(0.80-1.23) | 0.92(0.76-1.10) | 0.97(0.84-1.12) | 1.36(0.87-2.14 | 0.75(0.46-1.22) |
| 25-29 | Reference | Reference | Reference | Reference | Reference | Reference |
| 30-34 | 0.97(0.93-1.02) | 1.08(0.94-1.25) | 1.01(0.90-1.14) | 0.93(0.84-1.02) | 0.96(0.71-1.30) | 0.98(0.76-1.25) |
| 35-39 | 0.97(0.91-1.03) | 1.09(0.90-1.31) | 1.03(0.89-1.20) | 0.89(0.78-1.00) | 0.73(0.49-1.09) | 0.76(0.54-1.06) |
| 40-44 | 1.01(0.92-1.10) | 1.23(0.94-1.61) | 1.09(0.88-1.35) | 0.84(0.70-1.02) | 1.02(0.60-1.75) | 0.67(0.39-1.14) |
| 45+ | 1.07(0.94-1.22) | **1.68(1.16-2.44)^c^** | 1.02(0.74-1.40) | 0.80(0.60-1.06) | 0.89(0.42-1.85) | 1.05(0.51-2.15) |

a. Adjustment for linear and quadratic terms of maternal age, parental age difference, gender of the children, parity, calendar year of the children and maternal infection during pregnancy; b. No cases; c. P<0.05.
