# Supplementary material for: Paternal Age and Offspring Congenital Heart Defects: A National Cohort Study
Source: PLoS One. 2015 Mar 25;10(3):e0121030. doi: 10.1371/journal.pone.0121030 (PMC4373953; doi:10.1371/journal.pone.0121030)
Supplement: S3 Table — (DOCX) [file pone.0121030.s004.docx]

**S3 Table.** **Adjusted^a^ hazards ratio of CHDs and its five common subtypes among children with CHDs family history by different paternal age groups**

| Paternal age | CHD(n=988) | PDA(n=125) | ASD(n=173) | VSD(n=181) | TOF(n=29) | COA(n=29) |
| --- | --- | --- | --- | --- | --- | --- |
| <20 | **2.00(1.03-3.90)^c^** | **6.96(2.07-23.36) ^c^** | 4.28(0.93-19.68) | b | b | b |
| 20-24 | 1.17(0.91-1.50) | 0.79(0.34-1.82) | 1.42(0.71-2.83) | 1.16(0.63-2.13) | 2.14(0.55-2.30) | 1.71(0.46-6.33) |
| 25-29 | Reference | Reference | Reference | Reference | Reference | Reference |
| 30-34 | 1.00(0.84-1.20) | 1.26(0.75-2.12) | 1.33(0.86-2.08) | 1.05(0.69-1.61) | 0.77(0.29-2.07) | 1.38(0.51-3.70) |
| 35-39 | 0.97(0.77-1.23) | 1.62(0.85-3.07) | 1.00(0.57-1.76) | 1.12(0.66-1.90) | 0.44(0.10-1.20) | 0.55(0.11-2.75) |
| 40-44 | 1.14(0.82-1.59) | 1.69(0.66-4.28) | 1.30(0.61-2.78) | 1.12(0.52-2.40) | 1.86(0.30-11.50) | b |
| 45+ | 1.23(0.75-2.00) | 1.71(0.43-6.79) | 1.69(0.59-4.85) | 0.99(0.30-3.23) | b | b |

a. Adjustment for linear and quadratic terms of maternal age, parental age difference, gender of the children, parity, calendar year of the children and maternal infection during pregnancy; b. No case; c. P<0.05.
